# Multidirectional Pharma-Toxicological Study on *Harpagophytum procumbens* DC. ex Meisn.: An IBD-Focused Investigation

**DOI:** 10.3390/antiox9020168

**Published:** 2020-02-18

**Authors:** Lucia Recinella, Annalisa Chiavaroli, Maurizio Ronci, Luigi Menghini, Luigi Brunetti, Sheila Leone, Bruno Tirillini, Paola Angelini, Stefano Covino, Roberto Venanzoni, Gokhan Zengin, Simonetta Di Simone, Maria Chiara Ciferri, Viviana di Giacomo, Amelia Cataldi, Monica Rapino, Valentina Di Valerio, Giustino Orlando, Claudio Ferrante

**Affiliations:** 1 Department of Pharmacy, University “G. d’Annunzio” of Chieti-Pescara, 66100 Chieti, Italy; lucia.recinella@unich.it (L.R.); annalisa.chiavaroli@unich.it (A.C.); luigi.menghini@unich.it (L.M.); luigi.brunetti@unich.it (L.B.); sheila.leone@unich.it (S.L.); disimonesimonetta@gmail.com (S.D.S.); Mariachiara.ciferri@outlook.it (M.C.C.); viviana.digiacomo@unich.it (V.d.G.); amelia.cataldi@unich.it (A.C.); claudio.ferrante@unich.it (C.F.); 2 Department of Medical, Oral and Biotechnological Sciences, University “G. d’Annunzio” of Chieti-Pescara, 66100 Chieti, Italy; maurizio.ronci@unich.it; 3 Department of Biomolecular Sciences, University of Urbino, 61029 Urbino, Italy; bruno.tirillini@uniurb.it; 4 Department of Chemistry, Biology and Biotechnology, University of Perugia, 06100 Perugia, Italy; paola.angelini@unipg.it (P.A.); stefano.covino@unipg.it (S.C.); roberto.venanzoni@unipg.it (R.V.); 5 Physiology and Biochemistry Laboratory, Department of Biology, Science Faculty, Selcuk University, Campus, 42103 Konya, Turkey; 6 Genetic Molecular Institute of CNR, Unit of Chieti, “G. d’ Annunzio” University, Via dei Vestini 31, 66100 Chieti-Pescara, Italy; m.rapino@unich.it; 7 Department of Medicine and Ageing Sciences, “G. d’ Annunzio” University, Via dei Vestini 31, 66100 Chieti-Pescara, Italy; valentina.divalerio@unich.it

**Keywords:** *Harpagophytum procumbens*, IBDs, oxidative stress, inflammation, proteomic analysis

## Abstract

In the present study, we investigated the water extract of *Harpagophytum procumbens* DC. ex Meisn. in an experimental model of inflammatory bowel diseases (IBDs). Additionally, a microbiological investigation was carried out to discriminate the efficacy against bacterial and fungal strains involved in IBDs. Finally, an untargeted proteomic analysis was conducted on more than one hundred colon proteins involved in tissue morphology and metabolism. The extract was effective in blunting the production of oxidative stress and inflammation, including serotonin, prostaglandins, cytokines, and transcription factors. Additionally, the extract inhibited the growth of *Candida albicans* and *C. tropicalis*. The extract was also able to exert a pro-homeostatic effect on the levels of a wide plethora of colon proteins, thus corroborating a protective effect. Conversely, the supraphysiological downregulation of cytoskeletal-related proteins involved in tissue morphology and antimicrobial barrier function suggests a warning in the use of food supplements containing *H. procumbens* extracts.

## 1. Introduction

Inflammatory bowel diseases (IBDs) are chronic, relapsing, and multifactorial pathologies of the colon, which show increased and unbalanced intestinal immune response to external stimuli [1,2,3,4]. As a consequence of this condition, colon mucosa produces numerous pro-inflammatory biomarkers, including reactive oxygen/nitrogen (ROS/RNS) species, prostaglandins, and cytokines, which reinforce the inflammatory status, thus causing tissue damage [4]. Currently, the first choice drugs for treating IBDs are aminosalycilates, glucocorticoids, immune-suppressants, and tumor necrosis factor (TNF)α inhibitors. Nevertheless, a wide plethora of patients (20–40%) experiences the lack of efficacy or side effects, thus highlighting the urgent need of novel therapies, which could both implementing the efficacy and reducing the incidence of side effects [5]. Plant-derived extracts have long been described as possessing the capability of contrasting IBD-related oxidative stress and inflammatory pathways [6,7]. In this regard, it is of noteworthy interest to treat inflammatory conditions through home-made extracts prepared from plants traditionally used by folk populations. These extracts, especially those prepared with traditional and biocompatible solvents (water, hydroalcoholic solutions) in the forms of infusions or decoctions, could not only represent efficacious and safe treatment in the folk populations, but also represent innovative approaches for improving and valorizing local botanical resources and productive chains.

In the present study, we further deepened the protective effects of the previously described water extract of *Harpagophytum procumbens* DC. ex Meisn., also known as devil’s claw, in an ex vivo experimental model, that is, isolated colon specimens exposed with *Escherichia coli* lipopolysaccharide (LPS) [8]. In particular, the water extract of *H. procumbens* was further assayed for the determination of plant secondary metabolites belonging to the classes of phenols and flavonoids, namely, gallic acid, resveratrol, catechin, and epicatechin, as well as the iridoid compound harpagoside. Harpagoside is considered the main responsible component of the therapeutic activity of the plant; therefore, the measurement of its extract content (not lower than 1.2% w/w) represents an evaluation of the qualitative standard described in the European Pharmacopoeia (Menghini et al., 2019). In addition, we further investigated the possible mechanisms of the water extract of *H. procumbens* on multiple inflammatory and oxidative stress pathways by measuring the production of colon serotonin (5-HT), prostaglandin (PG)E_2_, and 8-iso-PGF_2α_, as well as tumor necrosis factor α (TNFα), nuclear factor kappa B (NFκB), interleukin (IL)-6, and nuclear factor erythroid 2-related factor 2 (Nrf2) mRNA levels. The putative extract mechanism was also investigated through an untargeted proteomic analysis. In this regard, the proteomic investigation was carried out on a cluster of more than 100 proteins involved in colon cell morphology and metabolism. Finally, the extract antimicrobial activity was studied against *E. coli*, *S. aureus*, *P. aeruginosa*, *Candida albicans*, and *C. tropicalis,* which are known to be involved in IBDs [9,10,11,12].

## 2. Materials and Methods

### 2.1. Pharmacognostic Studies

#### 2.1.1. Plant Material and Extraction Procedure

*H. procumbens* DC. ex Meisn. plant material was purchased in a local market in Namibia and authenticated by Prof. Luigi Menghini, head of the chair in Pharmaceutical Botany at the Department of Pharmacy of “G. d’Annunzio” University (Chieti, Italy). The pharmacognostic description of plant material and the preparation of the extract through ultrasound-assisted method is fully described in the Supplementary Materials section.

#### 2.1.2. Phytochemical Profile

*H. procumbens* water extract was analyzed for its content in phenols and flavonoids through validated colorimetric methods. Total phenols and flavonoids were expressed as equivalents of gallic acid and rutin, respectively. The acid gallic, catechin, epicatechin, and resveratrol content was also evaluated through independent high performance liquid chromatography (HPLC)-fluorimetric analysis, whereas the harpagoside level was measured with HPLC-diode array (DAD) analytical methods. The antiradical activity was assessed through 1,1-diphenyl-2-picrylhydrazyl (DPPH) radical and β-carotene/linoleic acid assays. The detailed protocols related to analytical methods and colorimetric assays are described in published papers [8,13,14] and reported in extenso in the Supplementary Materials section.

### 2.2. Toxicological, Pharmacological, and Microbiological Studies

#### 2.2.1. Artemia Salina Lethality Bioassay

The cytotoxicity of *H. procumbens* extract was formerly studied with *Artemia salina* lethality bioassay, whose protocol is fully described in the Supplementary Materials section.

#### 2.2.2. Ex Vivo Studies

Wild type (C57/BL6) male mice (2.5 months old, weight 20–22 g) were housed in plexiglas cages (2–4 animals per cage; 55 × 33 × 19 cm) and maintained under standard laboratory conditions (21 ± 2 °C; 55 ± 5% humidity) on a 14/10 h light/dark cycle, with ad libitum access to water and food, 24 h/day throughout the study, with no fasting periods. Mice were fed with a standard rodent chow (Prolab RMH2500, PMI Nutrition International, Brentwood, MO, USA). Housing conditions and experimentation procedures were strictly in accordance with the European Community ethical regulations (EU directive no. 63/2010) on the care of animals for scientific research. According to the recognized principles of “Replacement, Refinement and Reduction of Animals in Research”, colon specimens were obtained as residual material from vehicle-treated mice randomized in our previous experiments approved by the Local Ethical Committee (‘G. d’Annunzio’ University, Chieti-Pescara, Italy) and Italian Health Ministry (authorization no. 885/2018-PR).

Isolated mouse colon specimens were collected and maintained in a humidified incubator with 5% CO_2_ at 37 °C for 4 h, in RPMI buffer with added bacterial LPS (10 μg/mL) (incubation period), as previously reported [15]. *H. procumbens* extract (100–1000 μg/mL) and harpagoside (12 μg/mL) were used as pharmacological stimuli. Their efficacy was evaluated in comparison with the reference drug sulfasalazine (2 µg/mL). PGE_2_ and 8-iso-PGF_2α_ levels (ng/mg wet tissue) were measured in tissue and cell supernatants by radioimmunoassay (RIA), as previously reported [16]. Additionally, tissue homogenates were assayed for the determination of 5-HT level (ng/mg wet tissue) through HPLC coupled to electrochemical detection [17]. Individual colons were also dissected for evaluating tumor necrosis factor α (TNFα), nuclear factor kappa B (NFκB), interleukin (IL)-6, and nuclear factor erythroid 2-related factor 2 (Nrf2) gene expression, as previously reported [15,18]. The comparative 2^−ΔΔCt^ method was used to quantify the relative abundance of mRNA and then determine the relative changes in individual gene expression (relative quantification) [19]. Finally, an untargeted proteomic analysis was carried out on tissue homogenate [20,21]. The detailed description of RIA, real-time PCR and mass spectroscopy analysis is reported in the Supplementary Materials section.

#### 2.2.3. Antimicrobial Susceptibility Testing

In vitro antimicrobial activity of *H. procumbens* extract was assessed against three bacterial strains, namely, *P. aeruginosa* (ATCC 15442), *E. coli* (ATCC 10536), and *S. aureus* (ATCC 6538), and two yeasts and filamentous fungi, namely, *C. albicans* (YEPGA 6183) and *C. tropicalis* (YEPGA 6184). Voucher microbial cultures are maintained in the PeruMycA culture collection of the Department of Chemistry, Biology and Biotechnology (University of Perugia, Italy) and are available upon request. The extract activity was evaluated in comparison with reference anti-bacterial and anti-micotic drugs, namely ciprofloxacin (Sigma-Aldrich, Milan, Italy) and fluconazole (Sigma-Aldrich, Milan, Italy), respectively. The detailed protocols were described in our previous papers and are enclosed as supplementary materials [22,23].

#### 2.2.4. Human Colon Cancer HCT116 Cell Culture

HCT116 human colon carcinoma cell line (ATCC CCL-247) was maintained in DMEM supplemented with 10% FBS and penicillin-streptomycin (100 µg/mL). Cells were grown at 37 °C in a humified atmosphere of 5% CO_2_ and treated with LPS 10 μg/mL and different concentrations of *H. procumbens* water extract (1–1000 μg/mL). Cell viability was measured by MTT (3 [4–dimethylthiazol-2yl]-2,5-diphenyl tetrazolium bromide) growth assay as recently described [20]. Cell number was quantified by the amount of tetrazolium reduction in viable mitochondria. Cultured cells were seeded into a 96-well plate at 3 × 103 cells/well and treated as described above. After 24 and 48 h, the cells were processed according to the manufacturer’s instructions, and the absorbance of each sample was detected at 570 nm. Three independent experiments were performed under the same experimental conditions.

### 2.3. Statistical Analysis

The analysis of variance (ANOVA) followed by Newman–Keuls multiple comparison test was used to evaluate the statistical significance between the pharmacological group. GraphPad Prism version 5.01 for Windows (GraphPad Software, San Diego, CA, USA) was used as statistical software in order to compare the means ± S.D. for each experimental group. Statistical significance was set at *p* < 0.05. The number of animals randomized for each experimental group was calculated on the basis of the “resource equation” *N* = (E + T)/T (10 ≤ E ≤ 20) [24].

## 3. Results and Discussion

In the present study, a multidirectional investigation was carried out on the water extract from *H. procumbens* root subjected to an ultrasound-assisted extraction procedure at 60 °C, in order to mimic the traditional home-made extraction procedure [25]. The HPLC-DAD quantitative analysis [8,13] confirmed the validity of the extraction method that, in agreement with European Pharmacopoeia, gave harpagoside yield in the range of 1.2–1.5% (w/w). This result was also consistent with our previous study aimed at optimizing and validating the use of the *H. procumbens* water extract prepared with an automatic instrumental and scaling-up procedure [8]. The colorimetric assays also revealed the presence of total phenols and flavonoids in appreciable amounts (Table 1), whereas the HPLC-fluorimetric fingerprint analysis identified the presence of four secondary metabolites, namely, gallic acid, catechin, epicatechin, and resveratrol (Table 2), whose levels were comparable with that of harpagoside. In this regard, the antiradical/antioxidant activities shown by DPPH and β-carotene/linoleic acid assays (Table 3) are consistent with the cluster of extract secondary metabolites revealed by HPLC analyses [20,23], thus suggesting potential multiple antioxidant/anti-inflammatory mechanisms.

**Figure 1:**
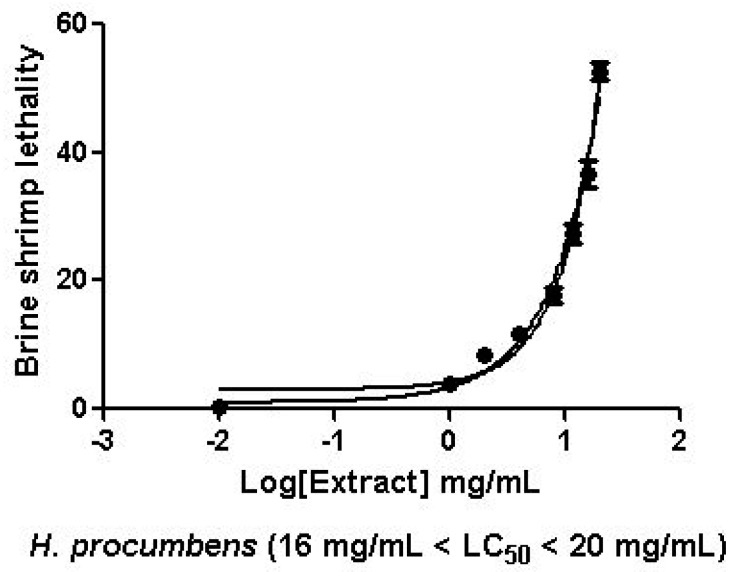
Effects of water *H. procumbens* extracts (0.1–20 mg/mL) on *Artemia salina* Leach viability (brine shrimp lethality test). Data are means ± SD of three experiments performed in triplicate.

**Figure 2:**
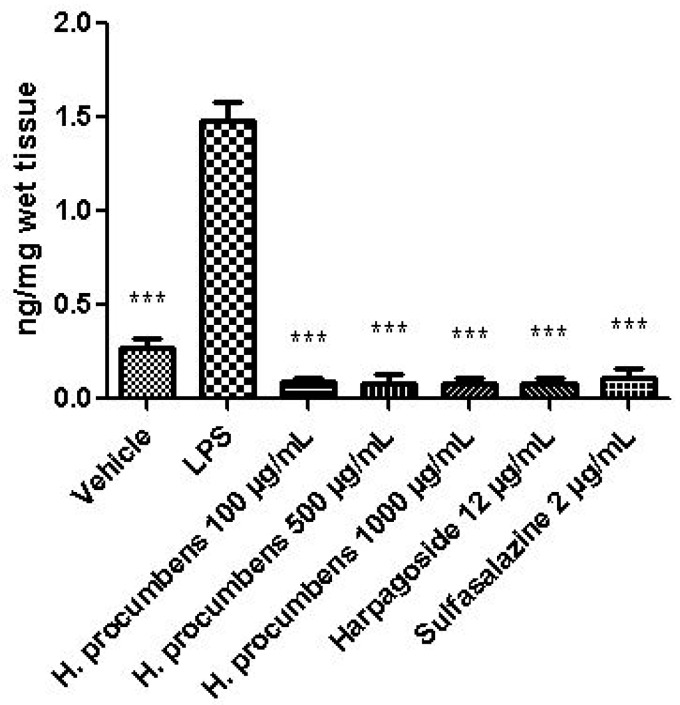
Effect of water *H. procumbens* extract (100–1000 µg/mL) on serotonin (5-HT) level (ng/mg wet tissue) in mouse colon specimens challenged with lipopolysaccharide (LPS). ANOVA, *p* < 0.0001; post-hoc, ***p < 0.001 vs. LPS.

**Figure 3:**
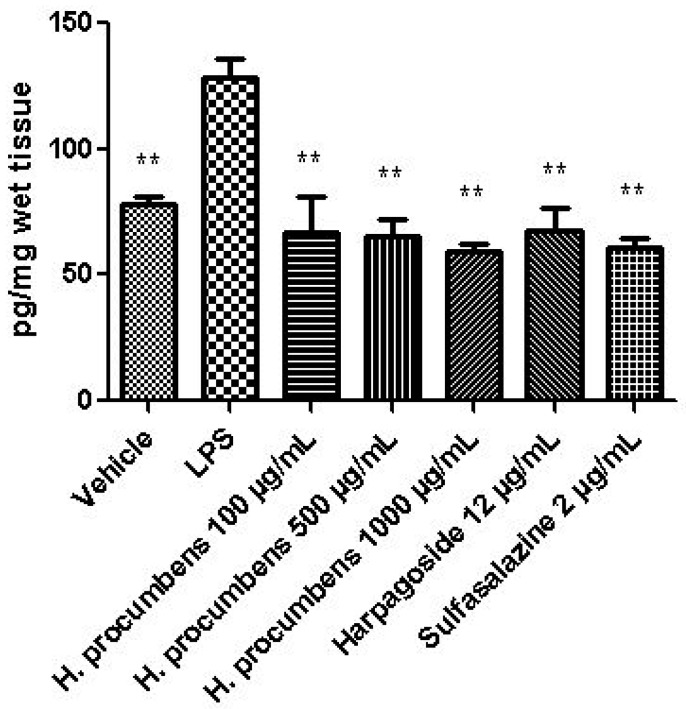
Effect of water *H. procumbens* extract (100–1000 µg/mL) on prostaglandin (PG)E_2_ level (pg/mg wet tissue) in mouse colon specimens challenged with LPS. ANOVA, *p* < 0.001; post-hoc, **p < 0.01 vs. LPS.

**Figure 4:**
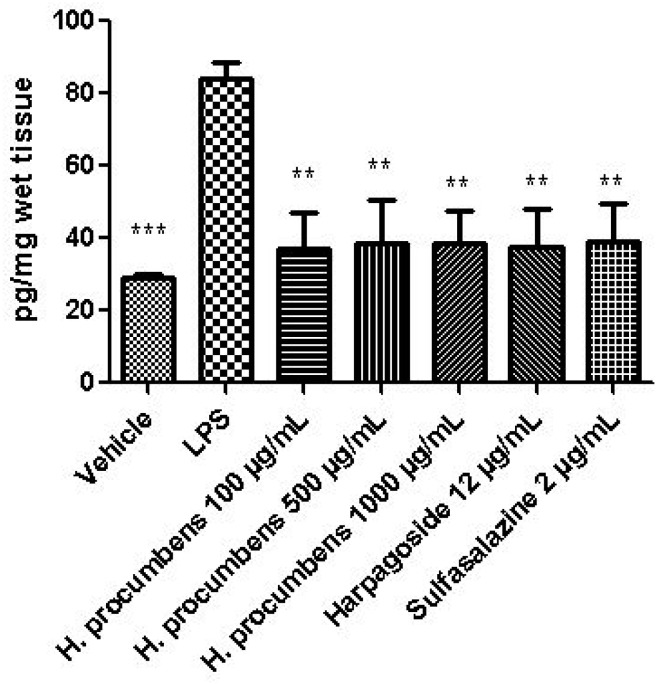
Effect of water *H. procumbens* extract (100–1000 µg/mL) on 8-iso-prostaglandin (PG)F_2α_ level (pg/mg wet tissue) in mouse colon specimens challenged with LPS. ANOVA, *p* < 0.001; post-hoc, **p < 0.01, ***p < 0.001 vs. LPS.

**Figure 5:**
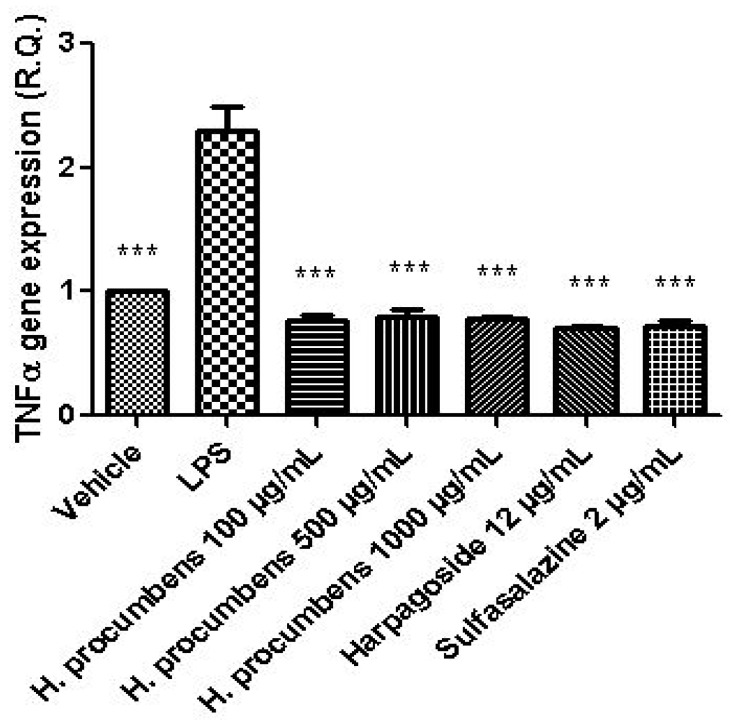
Effect of water *H. procumbens* extract (100–1000 µg/mL) on tumor necrosis factor (TNF)α gene expression (relative quantification) in mouse colon specimens challenged with LPS. ANOVA, *p* < 0.001; post-hoc, ***p < 0.001 vs. LPS.

**Figure 6:**
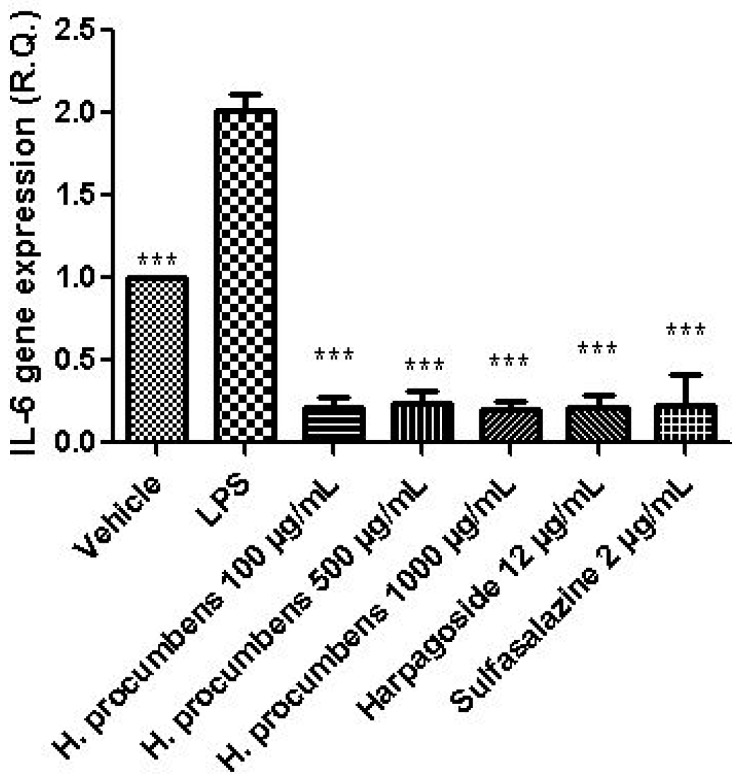
Effect of water *H. procumbens* extract (100–1000 µg/mL) on interleukin (IL)-6 gene expression (relative quantification) in mouse colon specimens challenged with LPS. ANOVA, *p* < 0.001; post-hoc, ***p < 0.001 vs. LPS.

**Figure 7:**
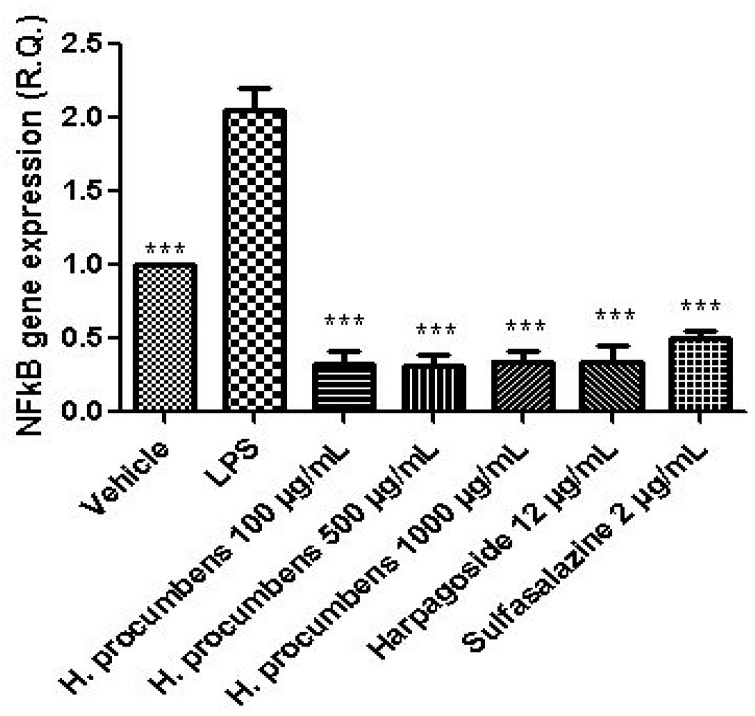
Effect of water *H. procumbens* extract (100–1000 µg/mL) on nuclear factor kappa B (NFκB) gene expression (relative quantification) in mouse colon specimens challenged with LPS. ANOVA, *p* < 0.001; post-hoc, ***p < 0.001 vs. LPS.

**Figure 8:**
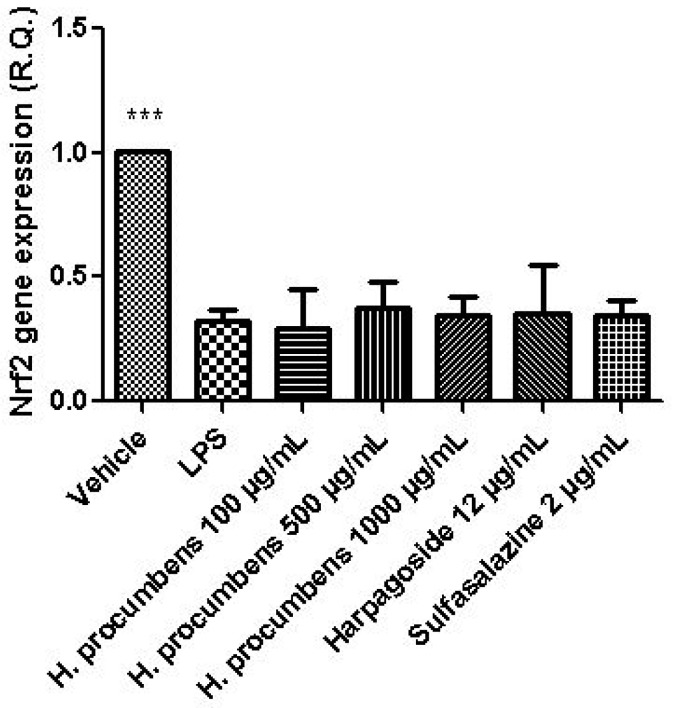
Effect of water *H. procumbens* extract (100–1000 µg/mL) on nuclear factor erythroid 2-related factor 2 (Nrf2) gene expression (relative quantification) in mouse colon specimens challenged with LPS. ANOVA, *p* < 0.001; post-hoc, ***p < 0.001 vs. LPS.

The extract was also tested on brine shrimp lethality assay, in order to verify the biocompatible limits for the subsequent pharmacological investigation. The brine shrimp *A. salina* Leach assay revealed an LC_50_ value > 10 mg/mL (Figure 1), thus corroborating the concentration range of 100–1000 μg/mL that was selected according to independent toxicological assays in our previous in vitro study [8]. The protective effects of the *H. procumbens* water extract (100–1000 μg/mL) was also evaluated on isolated mouse colon specimens exposed to LPS, in order to reproduce the burden of inflammation and oxidative stress occurring in ulcerative colitis, in vivo [26]. In particular, the extract effects were evaluated against the increased levels of pro-inflammatory and pro-oxidant colon mediators, including 5-HT, PGE_2_, and 8-iso-PGF_2α_. The mRNA levels of TNFα, IL-6, NFκB, and Nrf2 were measured, as well. As depicted in Figure 2, Figure 3 and Figure 4, the extract was able to blunt the LPS-induced level of 5-HT, PGE_2_, and 8-iso-PGF_2α_ in a concentration-independent manner. Additionally, the blunting effect was comparable to those exerted by both harpagoside (12 µg/mL) and sulfasalazine (2 µg/mL) used as the reference standard and drug, respectively. In particular, the employed concentration of harpagoside reflects its amount in the pharmacological group treated with the highest tested concentration of the extract (1000 μg/mL). In analogy, the extract exerted a concentration-independent inhibition of TNFα, IL-6, and NFκB gene expression (Figure 5, Figure 6 and Figure 7). Also in this case, the efficacy was comparable to those of harpagoside and sulfasalazine, whereas the extract was ineffective in reducing LPS-induced Nrf2 gene expression (Figure 8). Collectively, besides being in agreement with our previous study [8], the actual results suggest that the anti-oxidant and anti-inflammatory effects could not only be ascribed to the sole extract harpagoside content, but, albeit in part, to the other identified secondary metabolites. Particularly, the presence of gallic acid, catechin, epicatechin, and resveratrol is consistent with the reduced levels of the selected pro-oxidant/pro-inflammatory biomarkers [27,28,29]. Previously, Fiebich and colleagues [30] suggested that the anti-inflammatory *H. procumbens* activity could occur via the inhibition of the activator protein-1 (AP-1) transcription factor, without any involvement of NFκB and mitogen-activated protein kinase (MAPK) pathways. Nevertheless, the total phenolic and flavonoid content found in the extract could account for a possible involvement of both NFκB and MAPK pathways [27,29,31]. In this regard, our finding of reduced NFκB gene expression following extract treatment further supports the modulatory effect on multiple pathways controlling oxidative stress and inflammatory response. Actually, these discrepancies could be related, at least in part, to the differences in polarity and chemical composition between the described *H. procumbens* water extract and the hydroalcoholic (60% v/v) extract tested in Fiebich’s study. The water extract was also assayed against bacterial and fungal strains involved in colon inflammation, including *E. coli, P. aeruginosa, S. aureus, C. albicans,* and *C. tropicalis*. Microbiome dysbiosis, a pathological condition characterized by decreased colon concentration of beneficial *Firmicutes, Bacteroidetes, Actinobacteria,* and *Verrucomicrobia*, with a concomitant increase of pathogen bacteria belonging to *Enterobacteraceae*, was found to be deeply related to colon inflammation [32], despite there still being no identification of causative relationship. Conversely, multiple studies suggested the involvement of pathogens such as adherent-invasive *E. coli*, *P. aeruginosa,* and *S. aureus* [9,10,33]. A pivotal role in colon inflammation could be also played by *C. albicans* and *C. tropicalis,* whose presence in colonic mucosa could aggravate colitis clinical symptoms [11,12]. The antimicrobial assays demonstrated that the water extract of *H. procumbens* was completely ineffective against the tested bacterial strains (Table 4). This result was not surprising and was in agreement with previous papers investigating the potential anti-microbial activity of water and alcoholic *H. procumbens* extracts [34,35]. Conversely, the anti-micotic activity (Table 5), particularly against *C. tropicalis*, is of noteworthy interest and suggests the potential use of this extract against the opportunistic fungal infections occurring in ulcerative colitis [11]. Actually, the inhibitory activity on these fungal strains could be related, at least in part, to the total phenolic and flavonoid content of this extract [36,37,38].

**Figure 9:**
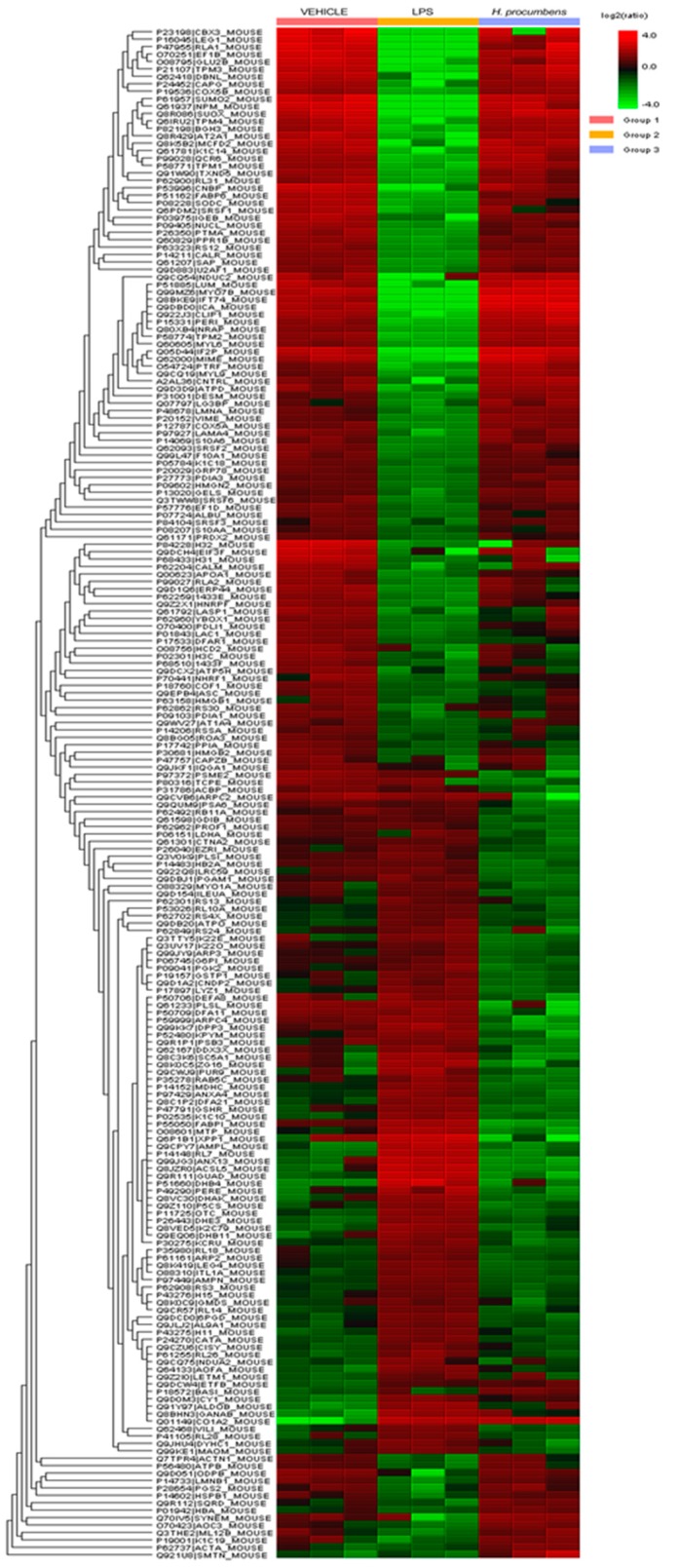
Untargeted proteomic analysis showing the effects of water *H. procumbens* extract (1000 µg/mL) on mouse colon specimens challenged with LPS. The activity of the detected proteins was calculated in comparison with the calibrator of the experiment (LPS group). In the figure, the green bar indicates a down-regulating effect compared to LPS, whereas the red bar indicates an up-regulating effect.

An untargeted and validated proteomic analysis [20,39] was also performed in order to not only deepen our knowledge about the protective effects exerted by *H. procumbens* water extract, but also to identify and/or predict potential and unknown collateral/adverse effects. In a second set of experiments conducted on colon specimens challenged with LPS, we evaluated the effect of the extract, tested at the highest concentration (1000 μg/mL), on the level of a wide plethora of colon proteins (*N* > 100). The levels of the identified proteins were expressed relative to the LPS group (positive control) and are depicted in Figure 9. In the figure, the green bar indicates a down-regulating effect compared to LPS, whereas the red bar indicates an up-regulating effect. It was observed that extract treatment showed the ability to exert a pro-homeostatic effect on most of the quantified proteins. In particular, the extract blunted the alteration of protein level induced by LPS, thus restoring the physiological condition observed in the vehicle-treated group. Of note are the pro-homeostatic effect on the levels of peroxiredoxin-2 (PRDX2), glutathione reductase (GSHR), catalase (CATA), and superoxide dismutase (SODC), which are deeply involved in contrasting oxidative stress-induced organ injury [40,41]. In addition, the water extract of *H. procumbens* normalized the levels of specific proteins, namely, drebrin-like protein (DBNL), macrophage-capping protein (CAPG), prothymosin alpha (PTMA), and high mobility group protein B2 (HGMB2), which are involved in the anti-proliferative effect, T-cell regulation, and defense against opportunistic infections [42,43,44,45]. Collectively, the pro-homeostatic effect exerted by the extract on the selected proteins is consistent with the reported anti-radical/anti-oxidant, anti-inflammatory, and anti-micotic effects. Moreover, considering the involvement of CAPG in the colon cancer progression [45], the present proteomic analysis further corroborates the anti-proliferative effect exerted by *H. procumbens* water extract (1000 μg/mL) on the human colon cancer HCT116 cell line [8]. On the other hand, the tested extract was ineffective against a limited number (*n* = 4) of proteins, whereas it determined a supra-physiological alteration of about 30 proteins, whose levels were not modified by the LPS stimulus. In particular, our attention focused on a group of proteins, namely ezrin (EZRI), actin-related protein 2/3 complex subunit 4 (ARPC4), plastin-1 (PLSI), and smoothelin (SMTN), which are involved in tissue morphology through multiple regulatory functions on cytoskeletal formation [46,47,48]. The supra-physiological alteration of protein level exerted by extract treatment could be at the basis of potential morphological alterations of colon tissue. Additionally, the extract was able to reduce the physiological concentration of colon α-defensin 8 (DEFA8) and α-defensin 11 (DEFA11). According to the putative role exerted by DEFA8 and DEFA11 in improving the function of the intestinal antimicrobial barrier (data reported in the recognized database “uniprot.org”), the supra-physiological downregulation of their levels after extract challenging could paradoxically lead to the onset of favorable conditions for the development of opportunistic pathogens, thus contrasting the observed intrinsic anti-microbial effects.

**Figure 10:**
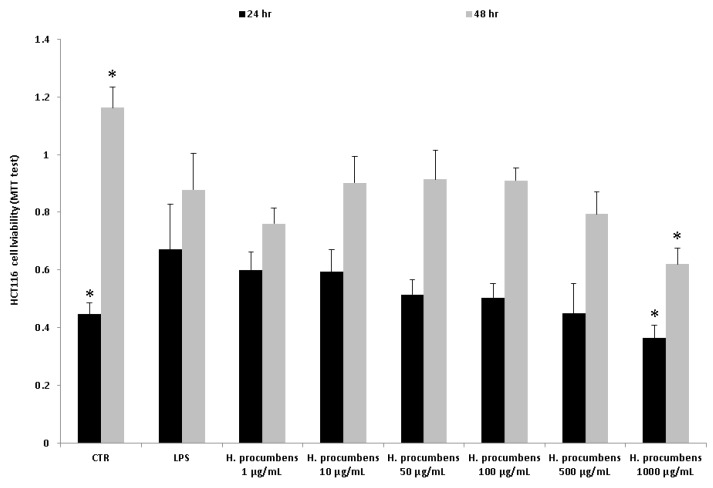
MTT assay of HCT116 human colon carcinoma cell line exposed to LPS 10 μg/mL and different concentrations (1–1000 μg/mL) of *H. procumbens* water extract for 24 and 48 h. ANOVA, *p* < 0.01; post-hoc, *p < 0.05 vs. LPS.

**Figure 11:**
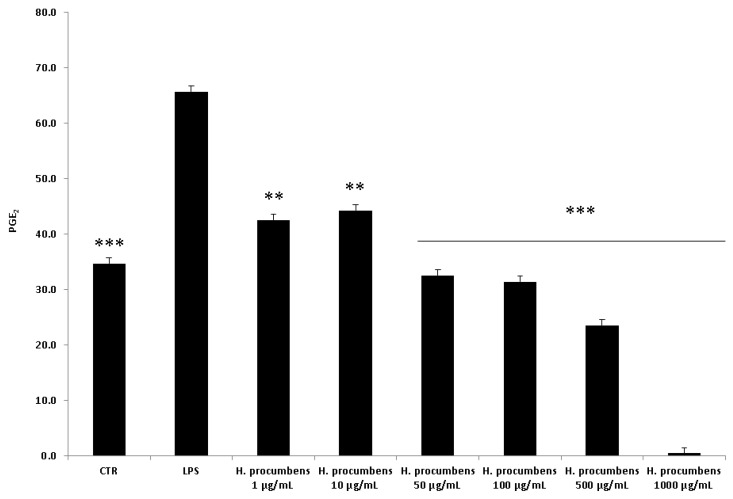
Effect of water *H. procumbens* extract (1–1000 µg/mL) on prostaglandin (PG)E_2_ level (pg/mL) in human colon HCT116 cells challenged with LPS. ANOVA, *p* < 0.0001; post-hoc, **p < 0.01, ***p < 0.001 vs. LPS.

**Figure 12:**
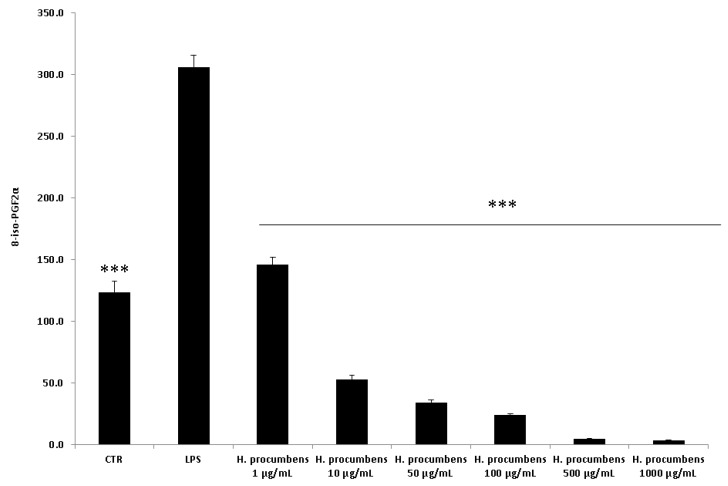
Effect of water *H. procumbens* extract (1–1000 µg/mL) on 8-iso-prostaglandin (PG)F_2α_ level (pg/mL) in human colon HCT116 cells challenged with LPS. ANOVA, *p* < 0.0001; post-hoc, ***p < 0.001 vs. LPS.

Finally, the concentration–response relationship was further investigated by challenging the HCT116 cells with the *H. procumbens* water extract at a wider concentration range (1–1000 µg/mL), compared to the aforementioned ex vivo tests. In this regard, the viability of HCT116 cells was evaluated in pro-inflammatory conditions at different time points (24–48 h). The treatment with LPS did not show cytotoxicity after 24 h, whereas it induced a decrease in cell viability after 48 h (Figure 10). On the other hand, the exposure to the highest concentration of *H. procumbens* water extract (1000 μg/mL) resulted in a decreased cell viability at both the experimental times. Furthermore, the release of PGE_2_ and 8-iso-PGF_2α_ was assessed. The results also showed a concentration-dependent inhibition of PGE_2_ and 8-iso-PGF_2α_ extracellular levels (Figure 11 and Figure 12). These results agreed with the antioxidant/anti-inflammatory effects observed in isolated colons, whereas the differences in the concentration–response relationship could have been due to different employed experimental models. In this regard, it is also noteworthy to highlight the efficacy of *H. procumbens* water extract in inhibiting hydrogen peroxide-induced ROS production from HCT116 cells, in a concentration-dependent manner, whereas the inhibition of cell viability, in basal condition, was significant at the highest tested concentration [8]. Overall, we hypothesize that HCT116 cells are more sensitive to the antioxidant/anti-inflammatory effects of the extract compared to isolated tissues, thus suggesting further research in order to explore potential chemo-preventive effects in colon cancer.

## 4. Conclusions

In conclusion, the present multidirectional study showed protective effects of *H. procumbens* water extract in reducing the burden of oxidative stress and inflammation in LPS-stimulated colon and HCT116 cells. Anti-microbial effects against pathogen fungal strains involved in IBDs were observed, as well. Additionally, the fingerprint phytochemical analyses suggested the involvement of multiple active principles, namely harpagoside, gallic acid, catechin, epicatechin and resveratrol in the observed pharmacological effects. Nevertheless, the supra-physiological downregulation of EZRI, ARPC4, PLSI, SMTN, DEFA8, and DEFA11 after extract treatment indicated potential morphological alterations in the colon tissue that should be taken in account in further researches. In this context, the present study recommends caution in the use of botanicals containing *H. procumbens.*

## Figures and Tables

**Table 1:** Total phenols and flavonoids determined via colorimetric assays of the *Harpagophytum procumbens* extract.

Table 1	Total Phenols		Total Flavonoids	
	mg/g Extract	SD	mg/g Extract	SD
*H. procumbens*	65.7	6.9	5.4	1.9

**Table 2:** Gallic acid, catechin, epicatechin, and resveratrol (mg/g) extract determined via HPLC-fluorimetric analysis of the *H. procumbens* extract.

Phenolic Content of *H. procumbens* Extract	mg/g Extract	SD
Gallic acid	9.74	0.88
Catechin	2.90	0.35
Epicatechin	3.03	0.18
Resveratrol	3.33	0.33

**Table 3:** Intrinsic antiradical activity of *H. procumbens* extract determined via colorimetric 1,1-diphenyl-2-picrylhydrazyl (DPPH) and β-carotene/linoleic acid assays.

Antitadical Activity	DPPH		β-Carotene/Linoleic Acid	
	IC_50_ µg/mL	SD	IC_50_ µg/mL	SD
BHT			2.5	0.32
Trolox	4.07	0.44	4.03	0.57
*H. procumbens*	121.01	16.6	16.8	2.05

**Table 4:** Antibacterial activity exerted by *H. procumbens* extract on *Escherichia coli, Pseudomonas aeruginosa,* and *Staphylococcus aureus.* * MIC values are reported as geometric means of three independent replicates (*n* = 3); MIC range concentrations are reported within brackets.

Table 4	MIC (µg/mL)*	
	*H. procumbens*	Ciprofloxacin
*E. coli* (clinical isolate)	11.80 (9.37–18.75)	<0.12
*P. aeruginosa* (clinical isolate)	188.98 (150–300)	<0.12
*S. aureus* (ATCC 6538)	>300	0.98

**Table 5:** Antimicotic activity exerted by *H. procumbens* extract on *Candida albicans* and *Candida tropicalis.* * MIC values are reported as geometric means of three independent replicates (*n* = 3); MIC range concentrations are reported within brackets.

Table 5	MIC (µg/mL)*	
	*H. procumbens*	Fluconazole
*C. albicans* (YEPGA 6183)	11.80 (9.37–18.75)	2
*C. tropicalis* (YEPGA 6184)	5.89 (4.68–9.37)	4

## Supplementary Materials

The following are available online at https://www.mdpi.com/2076-3921/9/2/168/s1, Supplementary Materials.

## Abbreviations

ARPC4	actin-related protein 2/3 complex subunit 4
BHT	butylated hydroxytoluene
CAPG	macrophage-capping protein
CATA	catalase
DBNL	drebrin-like protein
DEFA8	α-defensin 8
DEFA11	α-defensin 11
DPPH	1,1-diphenyl-2-picrylhydrazyl
EZRI	proteins namely ezrin
GSHR	glutathione reductase
HGMB2	high mobility group protein B2
5-HT	5-hydroxytryptamine, serotonin
IBDs	inflammatory bowel diseases
IL-6	interleukin-6
LPS	lipopolysaccharide
NFκB	nuclear factor kappa B
Nrf2	nuclear factor erythroid 2-related factor 2
PGE_2_	prostaglandin E_2_
8-iso-PGF_2α_	8-iso-prostaglandin-F_2α_
PLSI	plastin-1
PRDX2	peroxiredoxin-2
PTMA	prothymosin alpha
ROS/RNS	reactive oxygen/nitrogen species
SMTN	smoothelin
SODC	superoxide dismutase
TNFα	tumor necrosis factor α
